# Intraoperative vascular anastomosis occlusion due to cold agglutinin disease during brain surgery: a case report

**DOI:** 10.1186/s40981-025-00766-z

**Published:** 2025-01-18

**Authors:** Kazuma Kitamura, Mayumi Nakanishi, Naokazu Fukuoka, Kumiko Tanabe, Yoshinori Kamiya

**Affiliations:** Department of Anesthesiology and Pain Medicine, Gifu University Hospital, 1-1 Yanagido, Gifu, 501-1194 Japan

**Keywords:** Cold agglutinin disease, Vascular anastomosis, Brain surgery, Body temperature, Local warming

## Abstract

**Background:**

Cold agglutinin disease (CAD) is an autoimmune hemolytic anemia that induces blood coagulation and hemolysis upon exposure to cold temperatures. Strict temperature control is essential to mitigate these effects, especially during surgical procedures where hypothermia is possible.

**Case presentation:**

A 57-year-old male, 165 cm and 72 kg, diagnosed with CAD, underwent cerebral vascular anastomosis. Intraoperatively, mean arterial pressure was maintained at or above 65 mmHg with phenylephrine administration, while body temperature was rigorously controlled between 36.5 °C and 37.5 °C using forced-air warming blankets and heated intravenous infusions. Despite these measures, thrombotic occlusion occurred, necessitating surgical thrombus removal, intravenous heparin administration, and irrigation of the surgical field with warmed saline followed by re-anastomosis. The anastomosis remained patent without recurrence of thrombus formation thereafter.

**Conclusion:**

Preventing hypothermia is extremely important in the anesthesia management of CAD patients. However, careful attention must also be paid to temperature regulation in the surgical field.

## Background

Cold agglutinin disease (CAD) is a rare form of autoimmune hemolytic anemia (AIHA), characterized by blood clotting and hemolysis upon exposure to cold temperatures. The cold agglutinins responsible for CAD, often referred to as “cold antibodies,” are typically immunoglobulin M (IgM) antibodies [1]. These agglutinins become active at peripheral sites at approximately 29 °C, binding to red blood cells and causing agglutination. Concurrently, complement proteins become activated and bind to the cold agglutinins. As these agglutinins move toward the core of the body, they are released from the red blood cells due to the elevated temperature, while complement activation continues [2]. Thrombosis commonly develops in peripheral regions prone to cooling, such as the fingers, toes, ears, and nose, resulting in acrocyanosis and potential necrosis [3]. Hypothermia, especially in the context of cardiopulmonary bypass, has been previously reported [4] as a complicating factor in cardiovascular surgeries. Although challenges during free flap revascularization have been documented [5, 6], no previous reports have noted complications during brain surgery. In this case, an anastomotic occlusion occurred during a right superficial temporal artery-middle cerebral artery (STA-MCA) bypass procedure in a CAD patient. Thrombus removal, intravenous heparin administration, and substituting room-temperature saline for warmed saline (38 ˚C) ensured maintained patency.

## Case presentation

Written informed consent was obtained from the patient for the publication of the following case. A 57-year-old male, 165 cm and 72 kg, was diagnosed with CAD following the onset of limb cyanosis and hematuria during snow removal 3 months before surgery. A month before surgery, he presented with left-sided paresis and dysarthria, was diagnosed with right internal carotid artery occlusion and cerebral infarction, and was scheduled for right STA-MCA bypass surgery. His medical history included CAD, hypertension, diabetes, and hyperuricemia. He had a 37-year smoking history (30 cigarettes per day) but had ceased smoking a month before surgery. Preoperative evaluation revealed no abnormalities on the chest X-ray or electrocardiogram. Laboratory tests showed elevated D-dimer levels (34.9 mg/dL), a cold agglutination titer of 65,536, a positive direct Coombs test, and a C3d direct globulin test (C3d DAT) was positive with C3b/C3d of 3 + , confirming cold agglutinin disease. The patient continued to take clopidogrel (75 mg/day) for thromboprophylaxis; however, the patient ceased taking it 2 days prior to surgery. No heparin substitution was performed. The anesthetic approach involved total intravenous anesthesia with propofol and remifentanil, continuous phenylephrine administration, and bolus ephedrine to maintain a mean arterial pressure of at least 65 mmHg. Body core temperature was measured at the urinary bladder and the target core temperature was maintained between 36.5 °C and 37.5 °C. To achieve this, the operating room temperature was set at 26 °C upon patient admission the patient was entirely covered with a forced-air warming system, and intravenous fluids were heated to 41 °C and administered via LEVEL1® HOTLINE®. Extremities were monitored for cyanosis throughout the procedure.

**Fig. 1:**
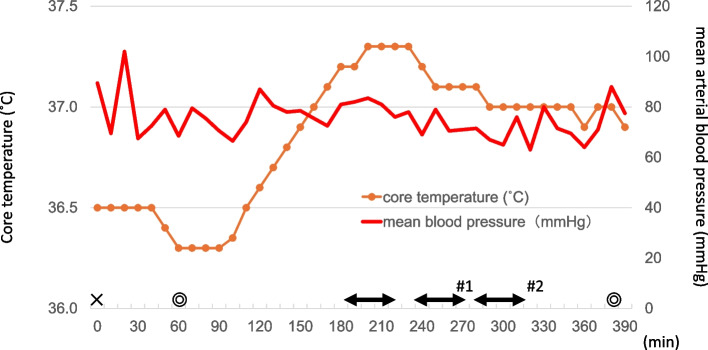
Changes in core (bladder) temperature and mean blood pressure over time. ⇔; Vascular anastomosis under a microscope. In #1, vascular occlusion of the anastomotic vessels due to a thrombus was observed, and thrombus removal was initiated. At the same time, heparin (3000 units) was administered intravenously, and the brain surface was irrigated with warmed (38 ˚C) saline. In #2, the activated clotting time was measured and was 178 s. ☓; start of the general anesthesia, ◎; start and end of the surgery

Core temperature was consistently maintained between 36.5 °C and 37.3 °C. Before starting the anastomosis, 200 mL of 20% mannitol was administered. The occlusion time of the vessel at the first anastomosis was 40 min with the shunt tube. Shortly after the second STA anastomosis, the surgeon detected an occlusion at the first anastomosis site by visual confirmation of a white thrombus (Fig. 1). The vessel was quickly incised, the occlusive thrombus was removed, and 3000 units of intravenous heparin were administered, extending the activated clotting time (ACT) to 178 s. Additionally, the saline used to irrigate the surgical field was replaced with warmed saline at 38 ˚C. After re-establishing the anastomosis, adequate blood flow to the MCA was confirmed using indocyanine green angiography, then the surgery was finished uneventfully. Postoperatively, the patient recovered without any new neurological deficits, and no signs indicative of cold agglutination, such as bilirubinuria, anemia, or peripheral cyanosis, were observed and were transferred to another hospital 8 days after surgery for rehabilitation.

## Discussion

CAD is a manifestation of AIHA. In CAD, IgM autoantibodies agglutinate erythrocytes in response to low temperatures and simultaneously activate the complement system, leading to extravascular hemolysis as erythrocytes are engulfed by reticuloendothelial cells expressing C3b receptors [1, 2]. Clinical manifestations include anemia due to erythrocyte agglutination and peripheral circulatory failure that can lead to life-threatening thrombotic complications due to microvascular occlusion [3]. It has been postulated that patients with CAD may be at risk for sudden death due to hemolysis or thrombotic events if they become hypothermic during surgery.

Anti-cold agglutinin antibody therapy may be an option to prevent thrombosis in CAD patients. Rituximab, an anti-CD20 monoclonal antibody, is used to suppress B cells that produce cold agglutinin antibody [7], and sutimlimab, an anti-C1s complement antibody, directly suppresses classical complement pathway-mediated hemolysis in patients by inhibiting the cleavage of C1s to C4 [8]. Moreover, plasma exchange may also be effective for red blood cell aggregation by cold agglutinin to remove IgM autoantibodies from plasm a [9]. However, these therapies have side effects of immunosuppression and extracorporeal circulation-induced hemolysis, and the procedure entails risks; it is crucial to consider the suitability of the method on a case-by-case basis.

While several case reports have detailed the anesthetic management of patients with CAD [4–6, 10, 11], particularly during cardiac surgery, most emphasize the importance of maintaining body temperature above 37 °C to avoid complications. The use of forced-air warming devices has been shown to be the most effective method of preventing hypothermia [12, 13]. Additionally, brain surgery allows for efficient maintenance of systemic warmth, as forced-air warming devices can cover the entire body. The patient’s core temperature was maintained by elevating the room temperature and employing forced-air warming systems, with no evidence of hemolysis. However, thrombosis occurred at the site of vascular anastomosis. In this case, irrigation with room-temperature saline may have induced anastomotic occlusion due to thrombus formation. This emphasizes the necessity of maintaining both systemic and local irrigation fluids at normothermic levels (37–38 °C).

In abdominal and thoracic surgery, peritoneal and pleural lavages are commonly performed with warmed saline to prevent hypothermia. There are also reports that adjusting the temperature of peritoneal lavage fluid can induce hypothermia [14] and accelerate recovery from hypothermia [15]. On the other hand, saline and buffer solutions used for local lavage of the brain surface during brain surgery are usually administered through an infusion line, so even if they are warmed, the temperature at the brain surface is equivalent to room temperature. In addition, from the perspective of brain protection, the brain surface temperature may be lower than body temperature, so they are often used without warming. Mechanical interruption of blood flow during anastomosis can cause thromboembolism. Although it is difficult to distinguish between CAD-induced and surgically induced thrombus formation, mechanical interruption of blood flow has not caused thrombus formation at the anastomosis site in previous STA-MCA bypass procedures performed at our institution. Therefore, it can be speculated that CAD was the main cause of thrombus formation in this case. However, in cases where there is a risk of thrombus formation when exposed to a low-temperature environment anywhere with blood flow, such as in this case, more careful consideration was required, such as using the brain surface lavage fluid in a warmed state. Furthermore, brain surface temperature monitoring, occasionally used in cases of severe head trauma, may prove beneficial in regulating the temperature of the surgical field to prevent cold agglutination, as observed in this case.

## Conclusion

We experienced a case of cerebrovascular anastomosis occlusion in a patient with CAD. Preventing hypothermia is extremely important in the anesthesia management of CAD patients. However, careful attention must also be paid to temperature regulation in the central nervous system and the surgical field.

## Data Availability

The data produced in this study are available from the corresponding author upon reasonable request.

## Abbreviations

CAD: Cold agglutinin disease
AHIA: Autoimmune hemolytic anemia
IgM: Immunoglobulin M
STA: Superficial temporal artery
MCA: Middle cerebral artery
ACT: Activated clotting time
